# Study on biomethane production and biodegradability of different leafy vegetables in anaerobic digestion

**DOI:** 10.1186/s13568-017-0325-1

**Published:** 2017-01-25

**Authors:** Hu Yan, Chen Zhao, Jiafu Zhang, Ruihong Zhang, Chunyu Xue, Guangqing Liu, Chang Chen

**Affiliations:** 1 Biomass Energy and Environmental Engineering Research Center, College of Chemical Engineering, Beijing University of Chemical Technology, 505 Zonghe Building, 15 North 3rd Ring East Road, Beijing, 100029 China; 2 College of Life Science and Technology, Beijing University of Chemical Technology, Beijing, 100029 China; 3 Department of Biological and Agricultural Engineering, University of California, Davis, CA 95616 USA

**Keywords:** Leafy vegetable, Composition, Anaerobic digestion, Correlation, Kinetic analysis

## Abstract

Enormous amounts of vegetable residues are wasted annually, causing many environmental problems due to their high moisture and organic contents. In this study, the methane production potential of 20 kinds of typical leafy vegetable residues in China were explored using a unified method. A connection between the biochemical components and the methane yields of these vegetables was well established which could be used to predict biogas performance in practice. A high volatile solid/total solid (VS/TS) ratio and hemicellulose content exhibited a positive impact on the biogas yield while lignin had a negative impact. In addition, three kinetic models were used to describe the methane production process of these agro-wastes. The systematic comparison of the methane production potentials of these leafy vegetables shown in this study will not only serve as a reference for basic research on anaerobic digestion but also provide useful data and information for agro-industrial applications of vegetable residues in future work.

## Introduction

In China, approximately seven hundred million tons of vegetables are produced annually, with two hundred million tons of residues and wastes. The accumulation of these residues may not only cause serious environmental problems but also lead to a significant waste of resources, as the high organic content of vegetable residues could make them a potential feedstock for renewable energy. Leafy vegetable wastes are a very important class of vegetable residues. They are produced in very large amounts in all the wholesale markets in the world and their landfill disposal is quite difficult because of their perishability (Di Maria et al. 2014; Scano et al. 2014). Therefore, an efficient solution to these issues is urgently needed.

Anaerobic digestion (AD) is an effective biochemical degradation method that is widely used for the treatment and energy recovery from many kinds of biomasses, especially agricultural products and agro-wastes. This approach has been proven to be a more outstanding method for disposing organic waste than other technology. Incineration of biomass and waste is still not fully accepted by the public opinion. And as alternative technologies, pyrolysis or gasification still does not represent a proven technology because only a limited number of full scale installations has been built (Appels et al. 2011). Compared with other techniques, AD has many economical, efficient, and environmentally friendly advantages, which make this technology applicable to industrial energy generation processes (Molino et al. 2013).

Thus far, many studies have been conducted on the AD of vegetables. Among the relevant studies, most have focused on comparing co-digestion using several vegetables and other feedstock (Molinuevo-Salces et al. 2010; Yao et al. 2014). Others have aimed at exploring the AD of mixed vegetables (Zhu et al. 2014). Only a few studies have compared the methanogenic potential of several types of vegetables, but there is no standard and universally recognized procedure for the determination of the biomethane yield in these researches. It is difficult to find any regularity because the methods were not consistent, so that the methane production potential of single leafy vegetables must be known in a uniform digestion condition. It is also necessary to establish relationships between components and methane yield, which can be used to predict biogas production performance in practice.

The purpose of this research is as follows: (1) to investigate the characteristics of 20 different types of leafy vegetable residues comprehensively, (2) to explore the methane production potential of 20 types of leafy vegetable residues in AD using a standard and unified digestion method, (3) to establish a connection between biochemical components and biogas production performance, and (4) to find a suitable kinetic model for describing the AD process of leafy vegetables.

## Materials and methods

### Feedstock

The twenty types of leafy vegetables in this study are commonly available in China, but some of them have not been previously evaluated for methane production. The Latin name, common name, and abbreviation of the feedstock are shown in Table 1. All of the vegetable residues were obtained from a vegetable market (Beijing, China) and were ground to a particle size of 2–4 mm using a grinder (JOYOUNG, China). The inoculum was anaerobic sludge collected from Beijing Donghuashan Biogas Station which only used pig manure as a substrate. The sludge was taken each 3 months and preserved at room temperature. The total solid (TS) and volatile solid (VS) of the sludge were measured to be 6.12 and 4.14%, respectively. The precipitate was used as the inoculum and added to the digester according to the F/I ratio, and the supernate of the sludge was removed before batch digestion. The TS and VS concentrations were measured using a standard method (Clesceri et al. 2012). The elemental compositions (C, H, N) were analyzed using an organic element analyzer (Vario EL cube, Germany). Oxygen contents were determined (Rincon et al. 2012) by assuming C + H + O + N = 99.5% (on a VS basis). The contents of cellulose, hemicellulose, and lignin were determined using an AMKOM 2000 fiber analyzer (AMKOM, USA) by measuring neutral detergent fiber (NDF), acid detergent fiber (ADF), and acid detergent lignin (ADL) (Van Soest et al. 1991). Soluble protein and non-structural carbohydrate contents were measured by the Bradford method (Barbosa et al. 2009; Silvério et al. 2012) and the DNS assay (Marsden et al. 2007; Miller 1959), respectively. Lipid contents were determined by Soxhlet extraction using diethyl ether as the solvent (Xu and Li 2012). The volatile fatty acids (VFAs) contents were measured by an Agilent 7890A gas chromatograph equipped with a flame ionization detector with nitrogen as the carrier gas, using a previously reported method (Li et al. 2013a).

**Table 1:** Latin name, common name, and abbreviation of feedstock

Latin name	Common name	Abbreviation in this study
*Brassica oleracea L. var. capitata L.*	Cabbage	V1
*Brassica pekinensis (Lour.) Rupr.*	Chinese cabbage	V2
*Mentha haplocalyx Briq.*	Mint	V3
*Allium sativum L.*	Young garlic shoot	V4
*Toona sinensis (Juss.) M. Roem.*	Chinese toon sprout	V5
*Andrographis paniculata (Burm.f.) Nees*	Common andrographis	V6
*Nepeta cataria L.*	Schizonepeta	V7
*Coriandrum sativum L.*	Coriander	V8
*Brassica oleracea* *L. var.italic* *Planch.*	Broccoli	V9
*Brassica campestris L. var. purpuraria L. H. Bailey*	Purple cabbage	V10
*Lactuca sativa L. var. ramosa Hort.*	Romaine lettuce	V11
*Spinacia oleracea* *L.*	Spinach	V12
*Chrysanthemum coronarium* *L.*	Crowndaisy chrysanthemum	V13
*Brassica oleracea L. var. botrytis L.*	Cauliflower	V14
*Oenanthe javanica (Bl.) DC.*	Celery	V15
*Lactuca sativa L.*	Lettuce	V16
*Amaranthus tricolor L.*	Amaranth wood	V17
*Ipomoea aquatica Forssk.*	Water spinach	V18
*Allium tuberosum Rottler ex Spreng.*	Leek	V19
*Scrophularia ningpoensis Hemsl.*	Summer radish	V20

### Methane production

Briefly, different leafy vegetables were tested in reaction bottles (total volume of 500 mL). The initial VS concentration for batch feeding was set to 5 g/L, and the feedstock to inoculum ratio was 1. Distilled water was then added to a working volume of 250 mL. All oxygen was discharged from the digesters by filling with nitrogen gas and the digesters were then sealed with a rubber plug. After that, the digesters were placed in an incubator at 37 °C for 25 days. All bottles were shaken manually for 1 min twice a day. Three parallel samples were used for each vegetable to ensure accuracy. Biogas yield was calculated according to our previous paper (Liu et al. 2015). Methane content was analyzed using a 7890A GC (Agilent, USA) equipped with a thermal conductivity detector, with helium as the carrier gas (Li et al. 2013c).

### Determination of biodegradability

To calculate the theoretical maximum methane production (MMP), two methods were applied in this research (Buswell and Mueller 1952; Li et al. 2013b), one of which was based on the elemental content, as shown in Eqs. (1) and (2):

1 $$ {\text{C}}_{n} {\text{H}}_{a} {\text{O}}_{b} {\text{N}}_{c} { + }\left( {n - \frac{a}{ 4} - \frac{b}{ 2}{ + }\frac{ 3c}{ 4}} \right){\text{H}}_{ 2} {\text{O}} \to \left( {\frac{n}{ 2}{ + }\frac{a}{ 8} - \frac{b}{ 4} - \frac{ 3c}{ 8}} \right){\text{CH}}_{ 4} { + }\left( {\frac{n}{ 2} - \frac{a}{ 8}{ + }\frac{b}{ 4}{ + }\frac{ 3c}{ 8}} \right){\text{CO}}_{ 2} { + }c{\text{NH}}_{ 3} $$

2 $$ {\text{MMP}}_{\text{ele}} \left( {\frac{{{\text{mLCH}}_{ 4} }}{\text{gVS}}} \right){ = }\frac{{ 2 2. 4\times 1 0 0 0\times \left( {\frac{n}{ 2}{ + }\frac{a}{ 8} - \frac{b}{ 4} - \frac{ 3c}{ 8}} \right)}}{{ 1 2n{ + }a{ + 16}b{ + 14}c}} $$

The second method for calculating the MMP was based on the organic composition, as shown in Eq. (3) (Rincón et al. 2012):

3 $$ {\text{MMP}}_{\text{org}} \left( {\frac{{{\text{mLCH}}_{ 4} }}{\text{gVS}}} \right) = \left( \begin{aligned} 373{\text{VFA}} + 496{\text{Protein}} + 1014{\text{Lipids}} + \hfill \\ 415{\text{Carbohydrates}} + 727{\text{Lignin}} \hfill \\ \end{aligned} \right)/100 $$

All of the compositions in Eq. (3) were calculated based on VS, including volatile fatty acids (as C_2_H_4_O_2_), proteins (as C_5_H_7_NO_2_), carbohydrates (as C_6_H_10_O_5_), and lignins (as C_10_H_13_O_3_). The biodegradability (BD) can be obtained from the highest cumulative methane yield from experiment (experimental methane yield, EMY) and the MMP through two methods, as described in Eqs. (4) and (5):

4 $$ {\text{BD}}_{\text{ele}} = \,{\text{EMY}}/{\text{MMP}}_{\text{ele}} $$

5 $$ {\text{BD}}_{\text{org}} = \,{\text{EMY}}/{\text{MMP}}_{\text{org}} $$

### Kinetic modeling

Various kinetic models have been widely used to describe the process of AD. In this study, three of them were chosen. The first model is the first-order kinetic model, expressed as Eq. (6) (Lo et al. 2010):

6 $$ B\,{ = }\,B_{0} [ 1 - {\text{exp(}} - kt ) ] $$

As shown in the equation, *B* represents the simulated cumulative methane yield (mL g_VS_^−1^), *B*_*0*_ refers to the simulated maximum cumulative methane yield (mL g_VS_^−1^) of the vegetable residues, and *k* and *t* denote the first-order rate constant (d^−1^) and digestion time (d), respectively.

Another equation, the modified Gompertz model (Syaichurrozi et al. 2013), is expressed as Eq. (7):

7 $$ B\,{ = }\,B_{0} \times \exp \left\{ { - \exp \left[ {\frac{{\mu {\text{e}}}}{{B_{0} }}(\lambda  - t) + 1} \right]} \right\} $$

where *B* represents the simulated cumulative methane yield (mL g_VS_^−1^), *B*_*0*_ means the simulated maximum cumulative methane yield (mL g_VS_^−1^), *μ* stands for the maximum methane production rate (mL g_VS_^−1^ d^−1^), *λ* refers to the lag phase time (d), *t* represents digestion time (d), and e is equal to 2.7183.

The last model is the MBPPSA model (Owamah and Izinyon 2015), as expressed in Eq. (8):

8 $$ B\,{ = }\,B_{0} [1 - \exp ( - kt)]^{n} { + }I_{0} $$

In this model, *B* represents the simulated cumulative methane yield (mL g_VS_^−1^), *B*_*0*_ refers to the simulated maximum cumulative methane yield (mL g_VS_^−1^), *k* means a constant of the model (d^−1^), *t* is digestion time (d), *n* stands for the number of data points, and *I*_*0*_ represents an inhibition/stability/feasibility determination factor.

### Data processing

All experiments were performed in triplicate. Microsoft Excel 2010 (Microsoft, USA) was used for data processing. Origin 8.0 (OriginLab, USA) was used for graphing and fitting.

## Results

### Characteristics of feedstock

The characteristics of the samples are shown in Table 2. Generally, leafy vegetables had low TS contents (2.83–11.19%), which corresponded to their high moisture contents. Mint (10.89%), young garlic shoot (10.50%), and coriander (11.19%) had higher TS contents compared with those of the other samples (below 10%). The VS/TS ratios were found to range from 69.15 to 94.76%. Nine types of leafy vegetables showed relatively high VS/TS ratios (over 85%), and usually a high organic content was beneficial for methane production. The C/N ratios were generally low, in the range of 6.65–11.79. Only purple cabbage (11.79) and romaine lettuce (10.04) had ratios higher than 10.

**Table 2:** Physicochemical analysis and elemental compositions of leafy vegetable residues

Samples	TS (%)	VS (%)	VS/TS (%)	C (%TS)	H (%TS)	O (%TS)	N (%TS)	C/N
V1	4.82 ± 0.02	4.35 ± 0.02	90.25	39.51 ± 0.12	5.75 ± 0.01	40.00 ± 0.13	4.54 ± 0.01	8.70
V2	3.47 ± 0.02	2.86 ± 0.03	82.42	35.66 ± 0.21	5.26 ± 0.01	35.73 ± 0.05	5.36 ± 0.01	6.65
V3	10.89 ± 0.05	9.45 ± 0.05	86.78	42.82 ± 0.13	5.81 ± 0.01	32.03 ± 0.07	5.68 ± 0.01	7.54
V4	10.50 ± 0.06	9.95 ± 0.05	94.76	44.58 ± 0.23	6.07 ± 0.02	39.11 ± 0.04	4.53 ± 0.01	9.84
V5	8.04 ± 0.09	7.26 ± 0.08	90.30	43.60 ± 0.15	6.24 ± 0.01	33.47 ± 0.02	6.54 ± 0.01	6.67
V6	4.96 ± 0.05	3.43 ± 0.05	69.15	35.06 ± 0.41	4.81 ± 0.01	24.60 ± 0.07	4.34 ± 0.00	8.08
V7	6.41 ± 0.04	4.83 ± 0.00	75.35	37.04 ± 0.06	5.23 ± 0.01	27.50 ± 0.11	5.20 ± 0.01	7.12
V8	11.19 ± 0.19	8.18 ± 0.17	73.10	37.88 ± 0.17	5.47 ± 0.01	24.60 ± 0.10	4.79 ± 0.01	7.91
V9	8.96 ± 0.07	8.10 ± 0.07	90.40	42.24 ± 0.23	6.32 ± 0.02	35.48 ± 0.06	5.91 ± 0.00	7.15
V10	6.89 ± 0.07	6.29 ± 0.07	91.29	41.61 ± 0.23	5.81 ± 0.03	39.88 ± 0.05	3.53 ± 0.01	11.79
V11	2.83 ± 0.01	2.47 ± 0.01	87.28	43.16 ± 0.13	5.58 ± 0.01	33.80 ± 0.06	4.30 ± 0.01	10.04
V12	7.22 ± 0.22	5.26 ± 0.15	72.85	31.04 ± 0.08	4.38 ± 0.01	33.17 ± 0.08	3.90 ± 0.02	7.96
V13	5.11 ± 0.06	3.86 ± 0.02	75.54	33.12 ± 0.15	4.77 ± 0.01	33.53 ± 0.09	3.74 ± 0.01	8.86
V14	5.69 ± 0.06	5.13 ± 0.05	90.16	41.49 ± 0.11	6.29 ± 0.01	35.83 ± 0.04	6.09 ± 0.01	6.81
V15	5.80 ± 0.06	4.28 ± 0.06	73.79	30.40 ± 0.08	4.60 ± 0.01	34.18 ± 0.11	4.25 ± 0.02	7.15
V16	6.94 ± 0.10	6.12 ± 0.11	88.18	38.97 ± 0.16	5.39 ± 0.01	38.89 ± 0.05	4.49 ± 0.03	8.68
V17	5.91 ± 0.24	4.29 ± 0.20	72.59	33.06 ± 0.10	4.78 ± 0.01	29.76 ± 0.03	4.62 ± 0.01	7.16
V18	5.52 ± 0.02	4.46 ± 0.02	80.80	36.28 ± 0.09	5.19 ± 0.01	34.35 ± 0.04	4.58 ± 0.01	7.92
V19	7.37 ± 0.05	6.17 ± 0.04	83.72	38.12 ± 0.13	5.62 ± 0.01	34.55 ± 0.06	5.01 ± 0.00	7.61
V20	4.13 ± 0.03	3.17 ± 0.01	76.76	35.55 ± 0.07	5.27 ± 0.02	30.44 ± 0.05	5.11 ± 0.01	6.96

The biochemical compositions of the samples are shown in Table 3. The contents of soluble proteins, VFAs, lipids, and non-structural carbohydrates were relatively low. The contents of proteins and VFAs were in the ranges of 0.12–1.35% and 0.23–3.01%, respectively. The lipid contents ranged from 1.02 to 5.51%, and the contents of non-structural carbohydrates were in the range of 0.01–6.53%. The ash contents were in the range of 5.24–30.78%. Table 3 demonstrated that the main organic components of leafy vegetables were structural carbohydrates, including hemicellulose (14.37–68.24%), cellulose (6.61–20.67%), and lignin (0.22–12.12%). The largest differences were reflected in the content of hemicellulose, which might influence methane production.

**Table 3:** Biochemical compositions of leafy vegetable residues (based on TS)

Samples	Proteins (%)	VFAs (%)	Lipids (%)	Structural carbohydrates			Non-structural carbohydrates (%)	Ash (%)
				Hemicellulose (%)	Cellulose (%)	Lignin (%)		
V1	1.04 ± 0.01	0.35 ± 0.00	2.11 ± 0.03	68.24 ± 1.86	13.52 ± 0.61	0.44 ± 0.00	0.29 ± 0.00	9.75 ± 0.02
V2	1.13 ± 0.00	1.04 ± 0.00	1.26 ± 0.01	60.22 ± 4.00	16.46 ± 1.63	0.22 ± 0.13	6.53 ± 0.05	17.70 ± 0.53
V3	0.42 ± 0.00	0.23 ± 0.01	5.51 ± 0.03	33.38 ± 0.18	16.62 ± 0.10	10.34 ± 0.11	2.42 ± 0.01	13.21 ± 0.12
V4	0.55 ± 0.00	0.78 ± 0.01	4.33 ± 0.01	62.28 ± 0.56	12.87 ± 0.93	3.86 ± 0.61	2.02 ± 0.00	5.24 ± 0.01
V5	0.56 ± 0.00	0.88 ± 0.02	5.07 ± 0.05	38.14 ± 3.47	17.27 ± 1.13	10.30 ± 0.55	0.03 ± 0.00	9.77 ± 0.04
V6	0.78 ± 0.01	0.76 ± 0.00	2.64 ± 0.02	50.97 ± 0.37	12.08 ± 0.05	5.76 ± 0.73	0.01 ± 0.00	30.78 ± 0.30
V7	0.15 ± 0.00	3.01 ± 0.03	3.52 ± 0.01	14.37 ± 2.06	19.53 ± 1.02	12.12 ± 1.56	0.02 ± 0.00	24.63 ± 0.45
V8	0.88 ± 0.01	0.80 ± 0.00	5.35 ± 0.05	31.95 ± 3.18	14.55 ± 0.34	5.96 ± 0.50	0.35 ± 0.00	26.88 ± 0.27
V9	0.71 ± 0.00	0.34 ± 0.01	2.98 ± 0.01	54.89 ± 3.53	14.43 ± 0.77	3.90 ± 0.69	0.31 ± 0.01	9.52 ± 0.05
V10	0.57 ± 0.00	0.30 ± 0.00	3.53 ± 0.03	61.54 ± 3.21	11.04 ± 5.81	5.26 ± 0.25	1.62 ± 0.02	8.76 ± 0.10
V11	1.35 ± 0.01	1.90 ± 0.02	4.79 ± 0.03	56.36 ± 0.50	14.08 ± 1.30	4.88 ± 1.33	0.16 ± 0.00	12.47 ± 0.08
V12	0.23 ± 0.00	0.59 ± 0.00	3.37 ± 0.03	24.85 ± 0.28	7.87 ± 0.89	9.43 ± 1.35	0.03 ± 0.00	27.19 ± 0.08
V13	0.12 ± 0.00	0.76 ± 0.00	2.14 ± 0.01	29.71 ± 5.15	18.95 ± 0.46	8.53 ± 1.40	0.02 ± 0.00	24.51 ± 0.38
V14	0.80 ± 0.00	1.01 ± 0.04	2.05 ± 0.01	47.51 ± 4.32	6.61 ± 1.18	3.87 ± 1.26	1.55 ± 0.02	9.83 ± 0.13
V15	0.53 ± 0.00	0.73 ± 0.00	1.51 ± 0.03	37.84 ± 2.43	19.22 ± 0.78	3.32 ± 1.57	0.07 ± 0.00	26.18 ± 0.29
V16	1.21 ± 0.00	0.69 ± 0.01	4.02 ± 0.05	35.45 ± 2.91	20.67 ± 1.12	6.16 ± 0.48	0.84 ± 0.01	11.83 ± 0.22
V17	1.28 ± 0.00	1.09 ± 0.00	1.02 ± 0.01	37.98 ± 2.82	10.50 ± 0.73	11.13 ± 1.69	0.02 ± 0.00	27.38 ± 0.34
V18	0.42 ± 0.00	0.48 ± 0.01	1.66 ± 0.05	41.05 ± 0.95	14.61 ± 1.61	10.42 ± 1.77	0.02 ± 0.00	19.11 ± 0.05
V19	0.80 ± 0.01	1.54 ± 0.00	2.71 ± 0.03	54.90 ± 4.60	13.61 ± 0.68	4.81 ± 0.70	0.36 ± 0.00	16.32 ± 0.02
V20	0.30 ± 0.00	1.10 ± 0.00	3.42 ± 0.05	36.91 ± 2.97	15.64 ± 0.38	4.41 ± 0.55	0.93 ± 0.00	23.17 ± 0.69

### Methane production

The methane production performance and biodegradability (BD) of leafy vegetables are shown in Table 4. The highest and lowest EMY values were observed from cauliflower (249.61 mL g_VS_^−1^) and schizonepeta (81.52 mL g_VS_^−1^), respectively. The MMP_org_ values ranged from 385.95 to 521.85 mL g_VS_^−1^, while the MMP_ele_ values were in the range of 364.13–538.65 mL g_VS_^−1^. Generally, the MMP_org_ and MMP_ele_ values were similar for each vegetable except coriander. The BD values were calculated based on the ratio of EMY to MMP. The results showed that the highest and lowest BD of the feedstocks were 60.57% (broccoli) and 18.79% (schizonepeta), respectively, based on organic composition. On an elemental basis, the highest BD was 55.77% (cauliflower) and the lowest was 16.76% (schizonepeta). In addition, feedstocks with high EMY values also showed relative high biodegradability.

**Table 4:** Methane production potential and biodegradability of the feedstock

Samples	EMY mL g_VS_^−1^	MMP_org_ mL g_VS_^−1^	MMP_ele_ mL g_VS_^−1^	BD_org_ %	BD_ele_ %	EMY’ mL g_VS_^−1^
V1	204.34	413.65	403.75	49.40	50.61	215.25
V2	129.34	449.07	393.73	28.80	32.85	131.65
V3	131.25	454.54	482.11	28.88	27.23	142.67
V4	227.88	436.75	447.51	52.17	50.92	241.38
V5	129.11	448.73	473.37	28.77	27.27	139.35
V6	118.04	521.85	508.20	22.62	23.23	198.40
V7	81.52	433.78	486.45	18.79	16.76	85.69
V8	199.61	443.31	538.65	45.03	37.06	179.76
V9	246.87	407.57	457.55	60.57	53.95	210.76
V10	232.70	446.68	429.78	52.10	54.14	240.91
V11	244.41	471.01	477.97	51.89	51.13	198.55
V12	157.90	385.95	376.24	40.91	41.97	157.55
V13	189.22	429.69	402.81	44.04	46.98	159.85
V14	249.61	463.62	447.55	53.84	55.77	237.73
V15	164.38	400.65	364.13	41.03	45.14	184.01
V16	199.07	403.74	400.65	49.31	49.69	198.89
V17	130.54	480.96	429.94	27.14	30.36	133.74
V18	171.50	458.87	418.14	37.37	41.01	148.18
V19	182.83	451.51	434.66	40.49	42.06	211.99
V20	183.51	407.17	447.93	45.07	40.97	212.60

### Correlation analysis

**Fig. 1:**
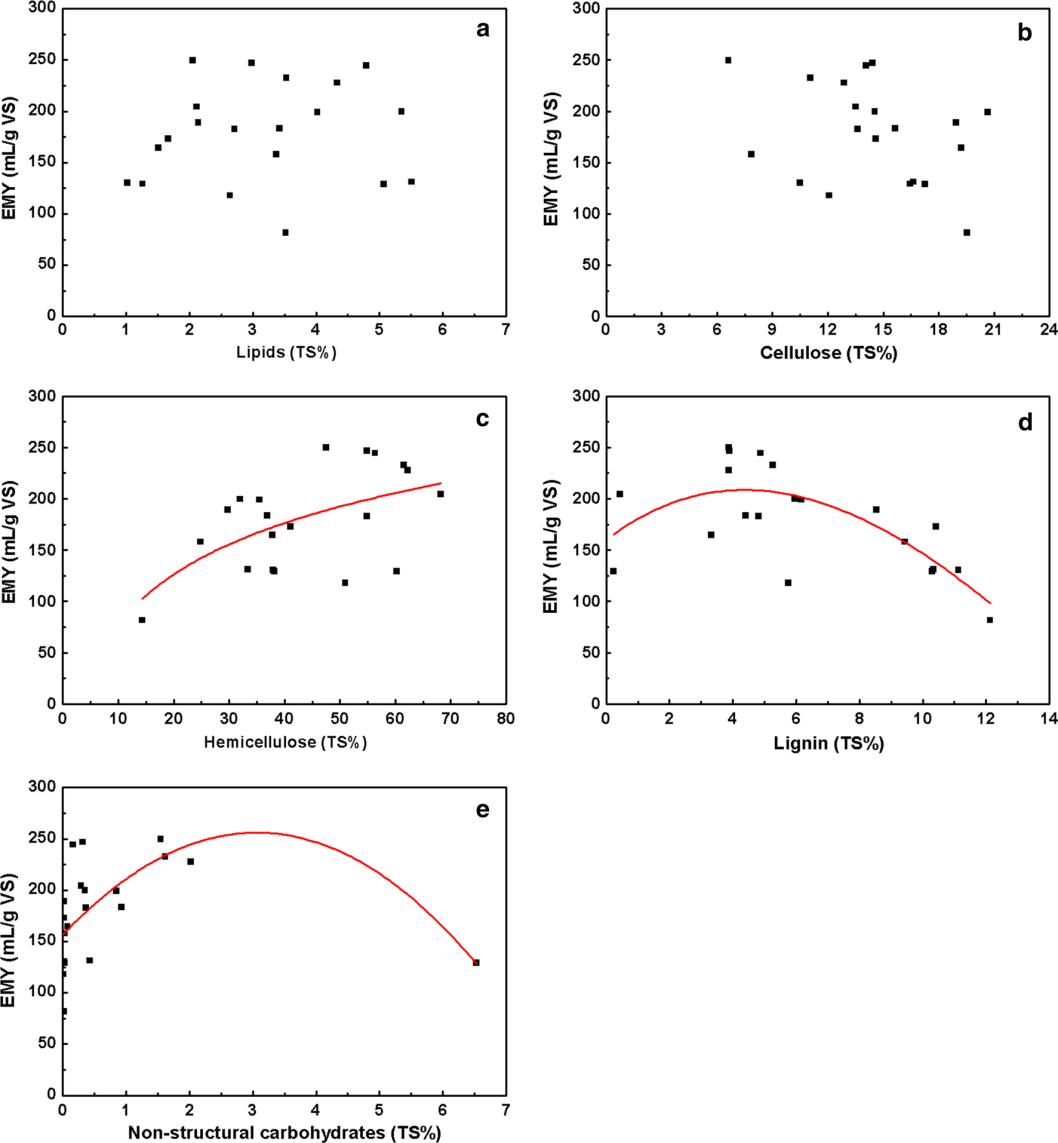
Correlation between lipids, hemicellulose, cellulose, lignin, and non-structural carbohydrates contents and experimental methane yield (**a**–**e**, respectively) of 20 kinds of leafy vegetables

Based on the biochemical compositions reported in Table 3, the appropriate functional forms of every organic component were determined based on differences in the regression coefficients (R^2^), and the results are shown in Fig. 1a–e. To explore the connection between EMY and the organic components, multiple linear regression analysis was applied. VFA and soluble protein levels were not considered as variables in the regression because their effects were proportionally small. Functional form for each organic component in the multiple linear regression model was determined through separate linear regression analysis. In addition, cellulose and lipid contents were found to have little influence in different forms, and no clear relations were observed with respect to EMY, thus, these forms were treated as simple functions.

According to the nonlinear curve fitting, a relational expression was discovered using the least squares method with the software EVIEWS (QMS, USA), as shown in Eq. (9):

9 $$ {\text{EMY}}^{\prime} = 8 2. 3 3- 2. 2 8 {\text{a}}^{ 2} + 1 3. 4 6 {\text{a}} + 2 8. 1 7 {\text{ln}}\left( {\text{b}} \right) - 0. 9 6 {\text{c}} - 0.0 7 {\text{d}}^{ 3} + 0. 3 9 {\text{d}}^{ 2} - 0. 4 3 {\text{d}} - 6. 4 7 {\text{e}}^{ 2} + 3 2. 5 4 {\text{e}} $$

In this formula, the variables are expressed as a percentage of TS. Variables a, b, c, d, and e refer to the contents of lipids, hemicellulose, cellulose, lignin, and non-structural carbohydrates, respectively. The EMY’ (simulated experimental methane yield) values of the feedstocks were calculated according to the equation (R^2^ = 0.913) and are shown in Table 4.

### Kinetic evaluation

**Fig. 2:**
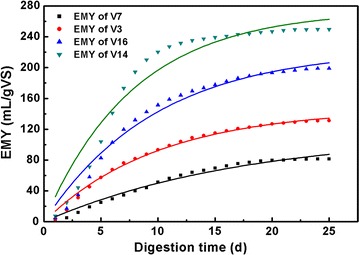
Results of nonlinear curve fitting of four representative leafy vegetables in first-order kinetic model

**Fig. 3:**
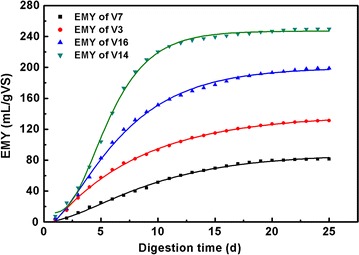
Results of nonlinear curve fitting of four representative leafy vegetables in modified Gompertz model

**Fig. 4:**
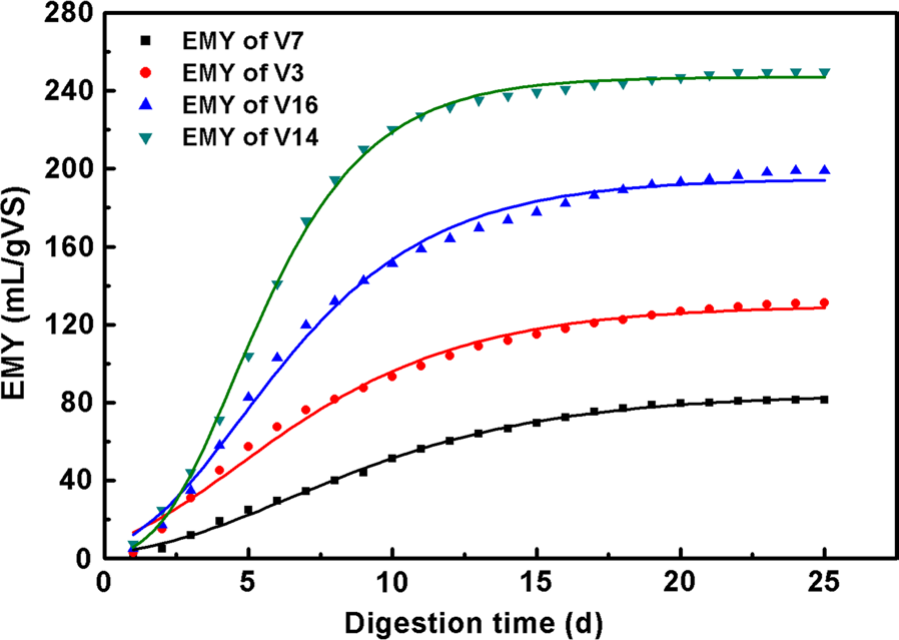
Results of nonlinear curve fitting of four representative leafy vegetables in MBPPSA model

The first-order, modified Gompertz, and MBPPSA models were used for kinetic evaluation, and the results are shown in Table 5. Four representative leafy vegetables (V3, V7, V14, V16) with different EMY values were chosen to show the difference among the three kinetic models, and the results are shown in Figs. 2, 3, 4. The parameters were all determined using non-linear regression through OriginPro 8.0 (OriginLab, USA) except *t*. Generally, R^2^ of the first-order kinetic model ranged from 0.794 to 0.990 which were lower than that obtained from the Gompertz kinetic model (0.985–0.999) and the MBPPSA kinetic model (0.995–1.000). The R^2^ values of three kinetic models indicated that the modified Gompertz model and MBPPSA model were more suitable for the AD of leafy vegetable residues than the first-order model. By comparing *B*_*0*_ (simulated maximum cumulative methane yield) and EMY, the *B*_*0*_ value of each type of vegetable was found to be relatively close to the EMY value in both the modified Gompertz and the MBPPSA model.

**Table 5:** Parameters in different kinetic models

Samples	First-order model			Modified Gompertz model				MBPPSA model					EMY mL g_VS_^−1^
	*B*_*0*_ mL g_VS_^−1^	*k* d^−1^	R^2^	*B*_*0*_ mL g_VS_^−1^	*μ* mL g_VS_^−1^d^−1^	*λ* d	R^2^	*B*_*0*_ mL g_VS_^−1^	*k* d^-1^	*n*	*I*_*0*_	R^2^	
V1	231	0.11	0.940	230	29.64	1.90	0.999	221	0.34	4.85	10.87	0.999	204
V2	147	0.10	0.973	130	12.98	1.12	0.994	140	0.20	1.84	−7.21	0.999	129
V3	147	0.10	0.990	141	11.62	0.18	0.990	168	0.13	1.01	−20.85	0.999	131
V4	232	0.09	0.897	267	28.34	4.68	0.997	262	0.28	9.76	4.93	0.998	228
V5	151	0.08	0.921	140	12.52	1.23	0.994	148	0.18	1.90	−4.45	0.998	129
V6	135	0.09	0.895	101	8.35	0.43	0.990	118	0.13	1.14	−15.25	0.999	118
V7	115	0.06	0.966	80	5.79	1.37	0.997	82	0.15	2.05	0.32	0.998	82
V8	219	0.12	0.821	179	21.05	1.25	0.993	189	0.25	2.18	−9.54	0.997	200
V9	272	0.12	0.810	277	33.67	1.38	0.999	273	0.29	3.08	5.58	0.999	247
V10	261	0.11	0.835	263	31.95	1.62	0.998	249	0.32	4.30	15.44	0.998	233
V11	277	0.11	0.857	265	29.88	1.54	0.999	263	0.27	3.09	4.53	0.999	244
V12	174	0.10	0.856	139	13.09	0.65	0.987	166	0.15	1.15	−26.42	0.997	158
V13	234	0.07	0.936	175	15.92	1.60	0.994	187	0.18	2.11	−8.07	0.998	189
V14	274	0.13	0.794	278	39.16	1.84	0.999	266	0.37	5.25	14.32	0.998	250
V15	179	0.11	0.841	147	14.32	0.54	0.985	175	0.16	1.13	−27.59	0.995	164
V16	224	0.10	0.870	215	21.51	1.05	0.994	225	0.20	1.88	−8.23	0.998	199
V17	154	0.08	0.916	116	10.53	1.20	0.990	130	0.17	1.59	−12.24	0.997	131
V18	191	0.10	0.860	170	16.89	0.90	0.992	187	0.19	1.57	−14.20	0.998	171
V19	201	0.12	0.823	189	21.11	1.10	0.998	191	0.25	2.39	0.57	1.000	183
V20	214	0.09	0.889	174	19.04	1.75	0.998	176	0.25	3.04	0.06	0.999	184

## Discussion

Generally, results from Tables 2 and 3 indicated that vegetables with high hemicellulose contents (over 55%) produced higher methane yields (over 200 mL g_VS_^−1^), with the exception of Chinese cabbage. Compared with other vegetables, schizonepeta showed poor potential for methane production, maybe because it had the lowest hemicellulose content and relative low VS/TS ratio among the vegetables. Poor methane yield has been also found for common andrographis, spinach, and amaranth wood, it might be owning to the low VS/TS. As well known, lignin content is another factor to influence the AD performance (Li et al. 2013b). It could be found that vegetable residues with a lignin content higher than 10% (V3, V5, V7, V17, V18), all possessed relatively low EMY. Collectively, the VS/TS ratio, lignin content, and hemicellulose content were important but not the only factors determining methane yield in anaerobic digestion.

In general, the BD and EMY values collectively determine whether a single substrate is suitable for AD to a certain extent. Through the analysis of the data in Table 4, substrates with higher EMY and BD were considered to have a good performance during AD tests. In addition, for the substrates with a low BD, certain pretreatment methods might make them more easily to be digested through the way of destroying the compact structure which is worthwhile to be researched in future.

It could also be found from Table 4 that the values of EMY’ and EMY were relatively close except for V6 (an outlier). A reliable connection between biochemical components and biogas performance has thus been established. The EMY values of leafy vegetables could be predicted well using this equation, which were considered to be more valuable and practical than the MMP. The results represented a worthwhile reference for examining the digestion of mixed leafy vegetables in further study.

In the modified Gompertz model, cauliflower showed the highest *μ* (maximum methane production rate) of 39.16 mL g_VS_^−1^d^−1^, and schizonepeta showed the lowest *μ* of 5.79 mL g_VS_^−1^d^−1^. A low value of *μ* corresponds to a low biogas production rate, which will eventually lead to poor biogas production. The *λ* (lag phase) values were all below 2 days except for the young garlic shoots (4.68 days), and a short lag time is preferred for AD. In the MBPPSA model, vegetables with higher *k* values (the constant of the model) showed better potential in methane production. The *I*_*0*_ values (the inhibition/stability/feasibility determination factor) ranged from −27.59 to 15.44. A negative value of *I*_*0*_ implied that the AD process was stable, while a positive *I*_*0*_ value indicated inhibition or instability. Normally, the higher the absolute negative and positive values of the *I*_*0*_ is, the more stable or instable the digester will be, respectively, however, it can not be concluded that good stability will result in high methane production.

In conclusion, after measuring the characteristics of 20 types of leafy vegetable residues and exploring their methane production potential with a simple and unified method, a dependent relationship was established between the EMY and the organic components to predict the AD performance. Results also showed that the VS/TS ratio, lignin content, and hemicellulose content exerted a combined influence on the methane yield. In addition, three kinetic models were used to evaluate the AD process of these agro-wastes. This research will not only serve as a reference for further study on the biogas production from different substrates but also contribute useful information for agro-industrial applications of vegetable wastes in the future. 

## Abbreviations

AD: anaerobic digestion
BD: biodegradability
TS: total solid
VS: volatile solid
MMP: theoretical maximum methane production
EMY: experimental methane yield
